# Three Autochthonous Cases of Amoebic Liver Abscess Clustered in a Small Village of Tuscany (Central Italy), a Non-Endemic Area

**DOI:** 10.3390/pathogens14070609

**Published:** 2025-06-20

**Authors:** Giuseppantonio Maisetta, Sara Moneta, Benedetta Tuvo, Cesira Giordano, Paola Alessandra Petrocelli, Giovanni Tincani, Daniela Campani, Davide Ghinolfi, Marco Falcone, Fabrizio Bruschi, Antonella Lupetti

**Affiliations:** 1 Department of Translational Research and New Technologies in Medicine and Surgery, Azienda Ospedaliero Universitaria Pisana, University of Pisa, 56126 Pisa, Italy; b.tuvo@studenti.unipi.it (B.T.); antonella.lupetti@unipi.it (A.L.); 2 Infectious Disease and Hepatology Unit, San Luca Hospital, 55100 Lucca, Italy; sara.moneta@uslnordovest.toscana.it; 3 Microbiology Unit, Azienda Ospedaliero Universitaria Pisana, University of Pisa, 56126 Pisa, Italy; cesira.giordano@ao-pisa.toscana.it; 4 Clinic Analysis Laboratory, San Luca Hospital, 55100 Lucca, Italy; paola.petrocelli@uslnordovest.toscana.it; 5 Division of Hepatic Surgery and Liver Transplantation, Azienda Ospedaliero Universitaria Pisana, University of Pisa, 56126 Pisa, Italy; g.tincani@ao-pisa.toscana.it (G.T.); d.ghinolfi@ao-pisa.toscana.it (D.G.); 6 Department of Surgical, Medical, Biochemical Pathology and Intensive Care, Azienda Ospedaliero Universitaria Pisana, University of Pisa, 56126 Pisa, Italy; daniela.campani@unipi.it; 7 Infectious Diseases Unit, Department of Clinical and Experimental Medicine, Azienda Ospedaliero Universitaria Pisana, University of Pisa, 56126 Pisa, Italy; marco.falcone@unipi.it; 8 Programma Monitoraggio delle Parassitosi e F.A.D., Azienda Ospedaliero Universitaria Pisana, University of Pisa, 56126 Pisa, Italy

**Keywords:** *Entamoeba histolytica*, liver abscess, abscess aspirate, molecular diagnosis

## Abstract

Amebiasis is a rare condition in industrialised countries but is epidemiologically growing. Clinical manifestations may range from asymptomatic to invasive disease. An amebic abscess can be the result of extraintestinal amebiasis, and it is associated with relatively high morbidity and mortality. We present three indigenous cases of amoebic liver abscesses observed within a few weeks (October–November 2023) in patients living in a small area near Lucca in Tuscany, Central Italy. Fever accompanied by abdominal pain and liver abscess was observed in all three patients, and one of them presented necrotising colitis and pleural effusion, too. The parasitological diagnosis was performed by microscopy and confirmed with real-time PCR in liver abscess drainage fluid and stools.

## 1. Introduction

*Entamoeba histolytica* is a protozoan parasite belonging to the Amoebozoa phylum, *Entamoeba* class, *Entamoebida* order, *Entamoebidae* family, and genus *Entamoeba*, which, with *Entamoeba dispar and Entamoeba moshkowvskii,* constitutes a complex [1]. Infection is usually caused by the ingestion of cysts in food or water previously contaminated by human faeces [2]. In most low-income countries, mainly distributed in tropical regions, with poor sanitation standards, amoebiasis is endemic, while the majority of cases in industrialised countries are represented by immigrants from endemic areas and individuals travelling to endemic areas [3]. *Entamoeba histolytica* can exert its pathogenic effect with several mechanisms, such as phagocytosis, killing and trogocytosis (ingestion of small fragments of cells), on a number of epithelial intestinal or liver cells and immune system cells [4]. As a result of its invasive capacity, the parasite is also able to cause extraintestinal infections, most frequently affecting the liver. Diarrhoea, fever, and right upper quadrant pain are the most common symptoms, but cough, chest pain, and weight loss can also be seen [5]. The amebic trophozoites in the liver cause inflammation and necrosis, leading to the formation of abscesses [6]. The amoebic liver abscess (ALA) diagnosis in a setting where amoebiasis is not endemic relies on a patient’s history, imaging (ultrasound imaging or a computerised tomography scan), serological tests, and analysis of a percutaneous fine-needle aspiration biopsy of the liver lesion [7,8,9]. A delay in diagnosis and treatment may cause fatalities. Here we report three clinical cases of ALA observed within two months and located in a small geographic area near Lucca in Tuscany (Central Italy).

## 2. Case Report 1

**Figure 1:**
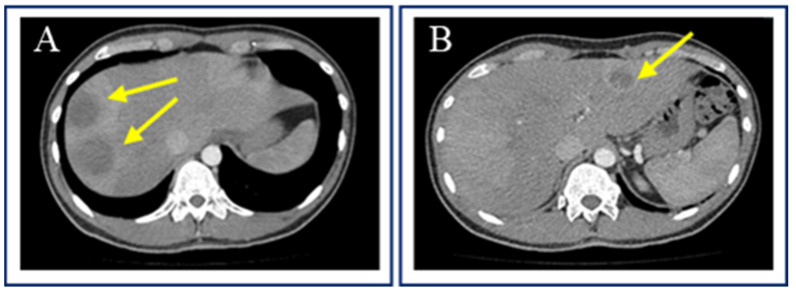
Abdominal CT scan analysis. Panel (**A**): two abscesses in the right hepatic lobe. Panel (**B**): an abscess in the left hepatic lobe. The arrows indicate abscess cavities.

A 29-year-old Italian male was admitted to the emergency room with pain in the right shoulder and at the right hypochondrium as well as a low-grade fever. The patient reported a 3-week history of diarrhoea three months earlier (non-bloody). Chest x-rays were negative, whereas abdominal ultrasound investigation revealed hepatic foci. A contrast-enhanced Computed Tomography scan (CT scan) of the abdomen showed the presence of three hepatic parenchymal lesions (Figure 1). Thus, the two major hepatic lesions were submitted to an ultrasonography-guided percutaneous drainage. The hepatic drainage was submitted to multiplex real-time PCR (Seegene Allplex GI-Parasite Assay; Seegene^®^, Seoul, Republic of Korea). DNA extraction was performed using the automated device STARlet (Hamilton Company, Reno, NV, USA) with the STARMag 96 Universal Cartridge kit, following the manufacturer’s instructions. Abscess samples were processed following the guidelines provided by the manufacturer. Approximately 1 g of abscess material was mixed with 2 millilitres of eNAT medium. The mixture was vortexed thoroughly and allowed to incubate at room temperature for 10 min to facilitate homogenisation. After incubation, 1 millilitre of the resulting suspension was transferred to a bead-beating tube and subjected to vigorous vortexing for 2 min. Genomic DNA was extracted using the Starlet automated extraction platform. The amplification of DNA was performed using a seven-plex PCR (Allplex^®^ GI parasite assay; Seegene^®^, Seoul, Republic of Korea). Amplification was realised on a CFX96 (Bio-Rad, Marnes-la-Coquette, France) and managed with CFX Manager IVD 1.6 software (Bio-Rad, Marnes-la-Coquette, France). The thermic protocol used for 44 amplification cycles was the following: 50 °C for 20 min, 95 °C for 12 min, 95 °C for 10 s, 60 °C for 1 min, 72 °C for 30 s. The hepatic drainage was positive for *E. histolytica* by multiplex real-time PCR, while negative for bacterial and fungal cultures. The light microscopy observation of fresh stool did not reveal the presence of *Entamoeba*, whereas multiplex real-time PCR analysis on stool was positive. After the parasitological diagnosis, the patient was treated for 3 weeks with ceftriaxone (2 g IV every 24 h), and metronidazole (750 mg IV every 8 h). At discharge, *E. hystolytica* using real-time PCR was absent in faeces; nevertheless, the patient was treated with paromomycin (750 mg OS every 8 h) for seven days to eliminate any residual intestinal amoebic trophozoites. The abscess lesions were almost resolved at the ultrasound control two weeks after discharge.

## 3. Case Report 2

**Figure 2:**
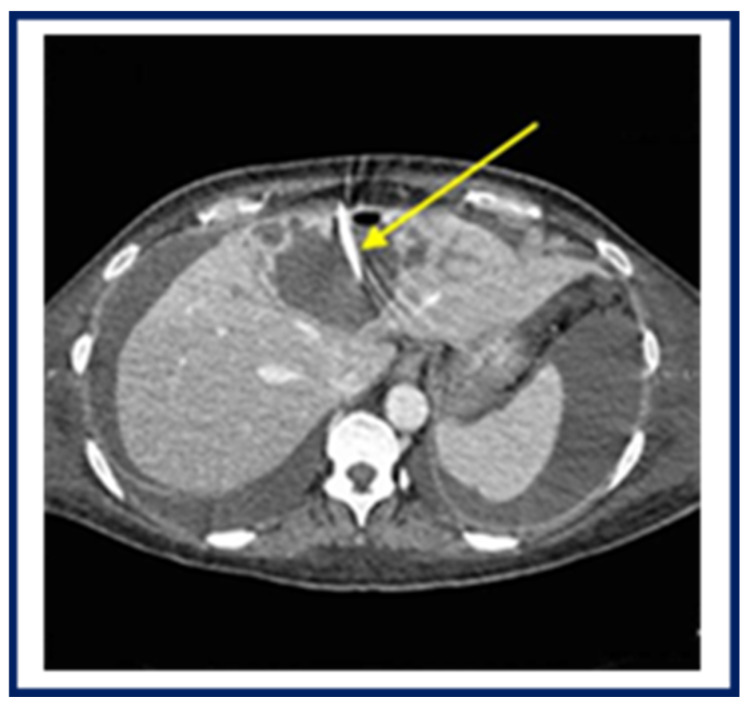
Abdominal CT scan of amoebic liver abscess before surgery. The arrow indicates a large abscess cavity and drainage at the centre of the liver.

A 49-year-old Italian female was admitted to the emergency room (Lucca Hospital) for an unresponsive fever to the treatment based on amoxicillin–clavulanate and corticosteroids. Due to abdominal pain, the patient underwent an abdominal CT scan, which showed the presence of a central hepatic cavity compatible with *Echiconococcus* cysts; however, an indirect chemiluminescent immunoassay (Virclia, Vircell, Granada, Spain), used for the search of anti-*Echinococcus granulosus* IgG, resulted negative. Due to the worsening of abdominal symptoms, the patient underwent positioning of percutaneous abdominal drainage under radiological guidance (Figure 2). Because of the onset of anaemia, several blood transfusions were performed. In light of the increased abdominal and pleural effusion, the patient underwent exploratory laparotomy surgery, peritoneal toilet and intrahepatic collection drainage.

**Figure 3:**
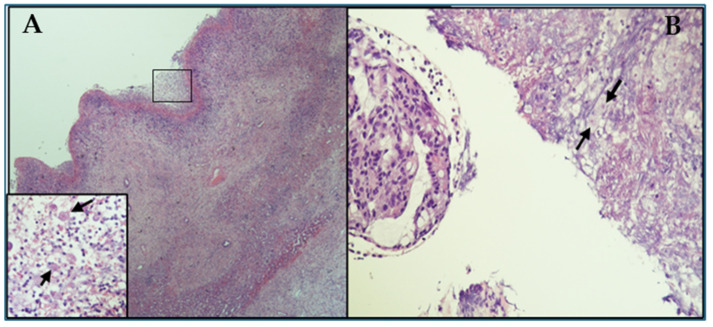
The liver and colon histological sections show amoebic trophozoites. The figure shows the patient’s liver and colon histological sections stained with haematoxylin–eosin. In Panel (**A**) a section of liver in which trophozoites are present in the abscess cavity is shown (200× magnification; the insert shows a detail at 400× magnification). In Panel (**B**) (400× magnification) a colon section in which many trophozoites are indicated by black arrows.

Intraoperative drainage fluid presented as brown necrotic material, resembling the classic amoebic “anchovy paste” appearance (Supplementary Figure S1A). Drainage fluid was analysed for bacteriological and parasitological analyses. Real-time PCR for protozoa detection yielded a positive result for *E. histolytica* (Supplementary Figure S2A). Cultures from both the abscesses and the peripheral blood ruled out the presence of bacterial/fungal infection. Multiplex real-time PCR (Allplex^®^ GI parasite assay; Seegene^®^, Seoul, Republic of Korea) performed on stool confirmed the positivity for *E. histolytica*. The histological examination of the liver sample confirmed the presence of hepatic fibrino-necrotic areas in association with several amoebic trophozoites (Figure 3A). Due to the appearance of rectal bleeding, a colonoscopy was performed, which highlighted a lesion in the cecum, on which biopsies were performed. Haematoxylin–eosin staining of colon histological sections showed several amoebic trophozoites associated with necrotic tissue (Figure 3B). The patient was treated with ceftriaxone (2 g IV every 24 h) and metronidazole (750 mg IV every 8 h). At discharge, the patient was treated with paromomycin (750 mg OS every 8 h) for seven days. The negative abdominal ultrasound and stool real-time PCR confirmed the patient’s complete recovery.

## 4. Case Report 3

**Figure 4:**
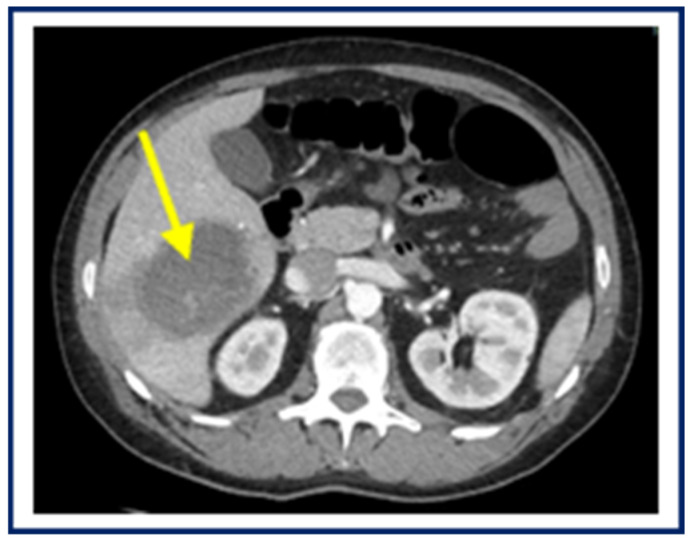
Abdominal CT of amoebic liver abscess before surgery. The arrow indicates the abscess cavity.

Due to worsening of abdominal pain, a 60-year-old Italian male underwent an abdominal ultrasound, which highlighted a liver abscess. The CT scan confirmed the presence of a liver abscess (maximum diameter 7.3 cm) (Figure 4).

The patient underwent ultrasound-guided percutaneous drainage (Supplementary Figure S1B), and the multiplex real-time PCR (Seegene Allplex GI-Parasite Assay) on drained material from the liver abscess resulted positive for *E. hystolitica* (Supplementary Figure S2B). The patient was treated for 3 weeks with ceftriaxone (2 g IV every 24 h) and metronidazole (750 mg IV every 8 h). After approximately 2 weeks, the surgical drainage was removed due to a clear reduction in the abscess fluid collection. The appearance of pulmonary embolism was reported, for which therapy with oral anticoagulants was started. At discharge, the multiplex-PCR for protozoa detection on the stool (Seegene Allplex GI-Parasite Assay) was negative; nevertheless, the patient was treated with paromomycin (750 mg OS every 8 h) for seven days. The main laboratory parameters of three patients at admission were reported in Supplementary Table S1.

## 5. Conclusions

Here we present three cases of invasive *E. histolytica* infections with liver abscesses. The three patients are Italian and live in a small village in Tuscany next to a little river. They did not refer to having travelled abroad, nor having had familial contacts among them, nor any other risk factor; Case 1 said that he usually eats fruit and vegetables from his garden that he irrigates with water from a nearby stream. This observation and the geographical proximity of the other two clinical cases support the hypothesis that the river could be the source of the infection. A molecular survey of 603 (out of 946 inhabitants) faecal samples from inhabitants in the same village of the three cases (Filecchio, Province of Lucca) detected two positive asymptomatic individuals (manuscript in preparation). Although cases of ALA have been recently described in Italy [10,11,12], to our knowledge, this is the first report describing a small infectious outbreak of amebic liver abscesses in Italy involving native patients. Interestingly, these three clinical cases of amoebic liver abscess were diagnosed thanks to a not-validated application of multiplex real-time PCR on the intraoperative drainage for rapid detection of the pathogen. Real-time PCR allowed us to perform a fast diagnosis and prompt the initiation of targeted therapy. Weitzel and colleagues described for the first time that real-time PCR might also serve as a rapid tool to diagnose amoebic liver abscess [13]. Our clinical cases and the recently described ALA cases in Italy indicate the importance of maintaining a high level of clinical vigilance for amebiasis, even in a non-endemic context.

## Data Availability

The original contributions presented in this study are included in the article/Supplementary Materials. Further inquiries can be directed to the corresponding authors.

## Supplementary Materials

The following supporting information can be downloaded at https://www.mdpi.com/article/10.3390/pathogens14070609/s1, Figure S1: Images of hepatic drainages; Figure S2: Amplification plots; Table S1: Main laboratory parameters of patients.
