# Supplementary material for: Three Autochthonous Cases of Amoebic Liver Abscess Clustered in a Small Village of Tuscany (Central Italy), a Non-Endemic Area
Source: Pathogens. 2025 Jun 20;14(7):609. doi: 10.3390/pathogens14070609 (PMC12299887; doi:10.3390/pathogens14070609)
Supplement: Supplementary file 1 [file pathogens-14-00609-s001.zip › pathogens-3700046-supplementary.pdf]

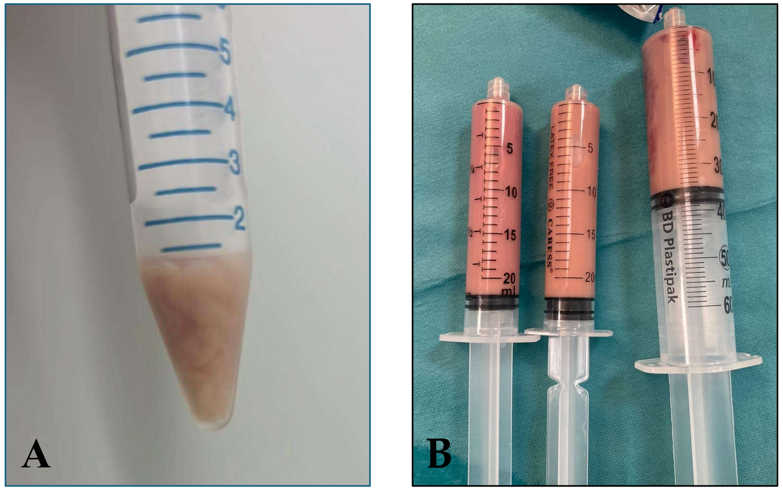

Figure S1 . Hepatic drainage of Case 2 (A) and Case 3 (B)

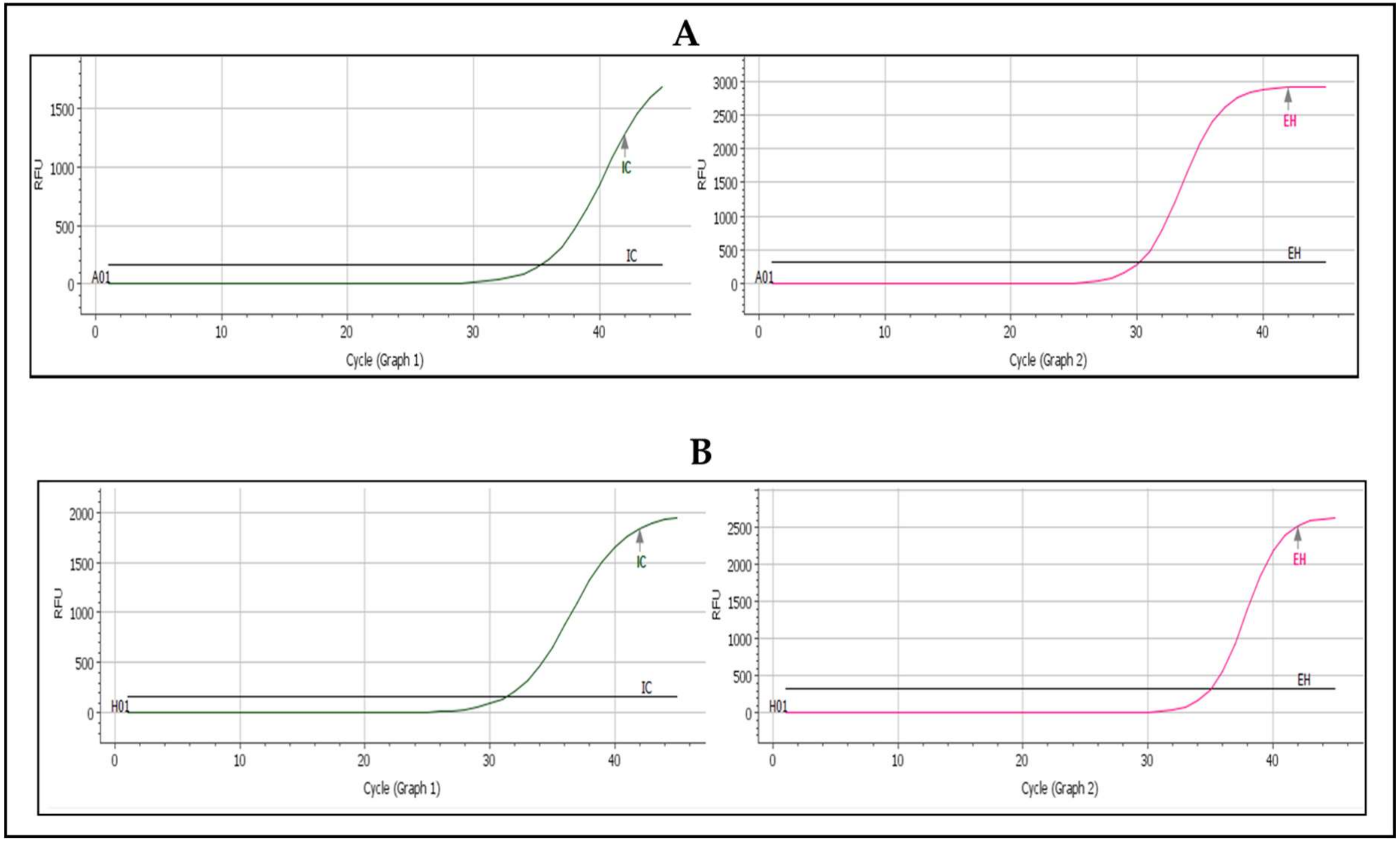

Figure S2: Amplification plot of Seegene Allplex GI-Parasite Assay for the Case 2 (A) and the Case 3 (B), indicating the presence of *Entamoeba histolytica*–specific DNA in abscess aspirate material. IC, internal control DNA.

	Leucocyte count /Neutrophil (%)	CRP (mg/dL)	ALT (U/L)	GGT (U/L)	ALP (U/L)
**Case 1**	$19x10^9$/L / 79%	25	51	N.V.	N.V.
**Case 2**	8.49 x 10^9 /L/ 73.6%	15.2	N.V.	284	376
**Case 3**	$13.9x10^9$/L / 83%	29	68	222	N.V.

Table S1. The table shows main patient laboratory parameters at admission. Legend: CRP: C-reactive Protein; ALT: Alanine amino-transferase; GGT: gamma-glutamyl transferase; ALP: alkaline phosphatase. N.V.: normal values.
